# Case Report: Clinical complete response of advanced renal cell carcinoma associated with Xp11.2 translocation/TFE3 gene fusion by treated by camrelizumab and axitinib: A rare case report

**DOI:** 10.3389/fphar.2022.927299

**Published:** 2022-08-11

**Authors:** Juping Zhao, Kun Dai, Jialing Xie, Chen Fang, Na Chen, Jun Dai, Danfeng Xu

**Affiliations:** ^1^ Department of Urology, Ruijin Hospital, Shanghai Jiao Tong University School of Medicine, Shanghai, China; ^2^ Hangzhou Jichenjunchuang Medical Laboratory Co.Ltd, Hangzhou, China; ^3^ Department of Pathology, Ruijin Hospital, Shanghai Jiao Tong University School of Medicine, Shanghai, China

**Keywords:** immunotherapy, PD-1, tyrosine kinase inhibitor, Xp11.2 translocation renal cell carcinoma, PTPRD-mutation

## Abstract

Renal cell carcinoma (RCC) associated with Xp11.2 translocation/TFE3 gene fusions is a rare subtype of renal tumor. This entity predominantly occurs in juveniles, but rarely in adults. Xp11.2 translocation RCC (tRCC) patients with lymph node or organ metastasis are associated with poor prognosis, and the strategy remains controversial. Herein, we presented our experience with the diagnosis and treatment of an adult case of Xp11.2 tRCC. In our clinical practice, a 32-year-old male manifested fever and right flank paroxysmal blunt pain, and computed tomography showed an inhomogeneous mass, 6 cm in diameter, in the right kidney. Then right partial nephrectomy (PN) and renal hilar lymph node dissection by laparoscopic surgery were performed. Pathology revealed that the tumor cells were positive for TFE3 immunohistologically and positive for TFE3 break-apart fluorescence *in situ* hybridization assay. A splice site mutation c.1544-1G>T of protein tyrosine phosphatase receptor delta (*PTPRD*) was detected by next-generation sequencing and weak PTPRD expression was confirmed in tumor tissues compared to tumor periphery. This patient was diagnosed with stage III RCC and received immune checkpoint inhibitor (camrelizumab) in combination with tyrosine kinase inhibitor (axitinib) treatment for 1 year. He achieved a clinical complete response with no sign of recurrence or metastasis. *PTPRD* mutation might be a favorable indicator for Xp11.2 tRCC patients managed by PN and followed by the adjuvant therapy of immune checkpoint inhibitor and tyrosine kinase inhibitor.

## Introduction

Xp11.2 translocation renal cell carcinoma (tRCC) is generally a pediatric renal cell carcinoma (RCC), accounting for 20%–40%, while relatively rare in adult RCC, only 1%–1.6% (Bruder et al., 2004; Kmetec and Jeruc, 2014). Xp11.2 tRCC was officially recognized as a distinct entity in 2004 and had been reported as being more aggressive and having a poorer prognosis compared with conventional RCC (Bruder et al., 2004; Qiu et al., 2010). This entity of RCC is generally characterized by a range of translocations on chromosome Xp11.2 leading to a gene fusion between TFE3 and at least six possible partners (Klatte et al., 2012; Sun et al., 2021a). The diagnosis of Xp11.2 tRCC is based on some immunohistochemical markers, such as TFE3, and fluorescent *in situ* hybridization (FISH) rather than histological characteristics and imaging examination (Calio et al., 2019). Although several studies revealed genomic and transcriptomic characteristics of this entity of RCC, the optimal strategy for Xp11.2 tRCC remains controversial. With its aggressive biological behavior and local invasion tendency in adults, Xp11.2 tRCC tends to present with lymphatic and distant metastasis at diagnosis. To date, there is not yet a consensus regarding therapy (Calio et al., 2019). Surgical resection achieved favorable outcomes for early disease (He et al., 2015; Liu et al., 2016). However, adult patients at the advanced stage had worse outcomes even with postoperative adjuvant monotherapy (He et al., 2015; Liu et al., 2016). We herein report a rare case with Xp11.2 tRCC treated with partial nephrectomy (PN) and followed by camrelizumab in combination with axitinib, with 18 month follow-up.

## Case presentation

**FIGURE 1:**
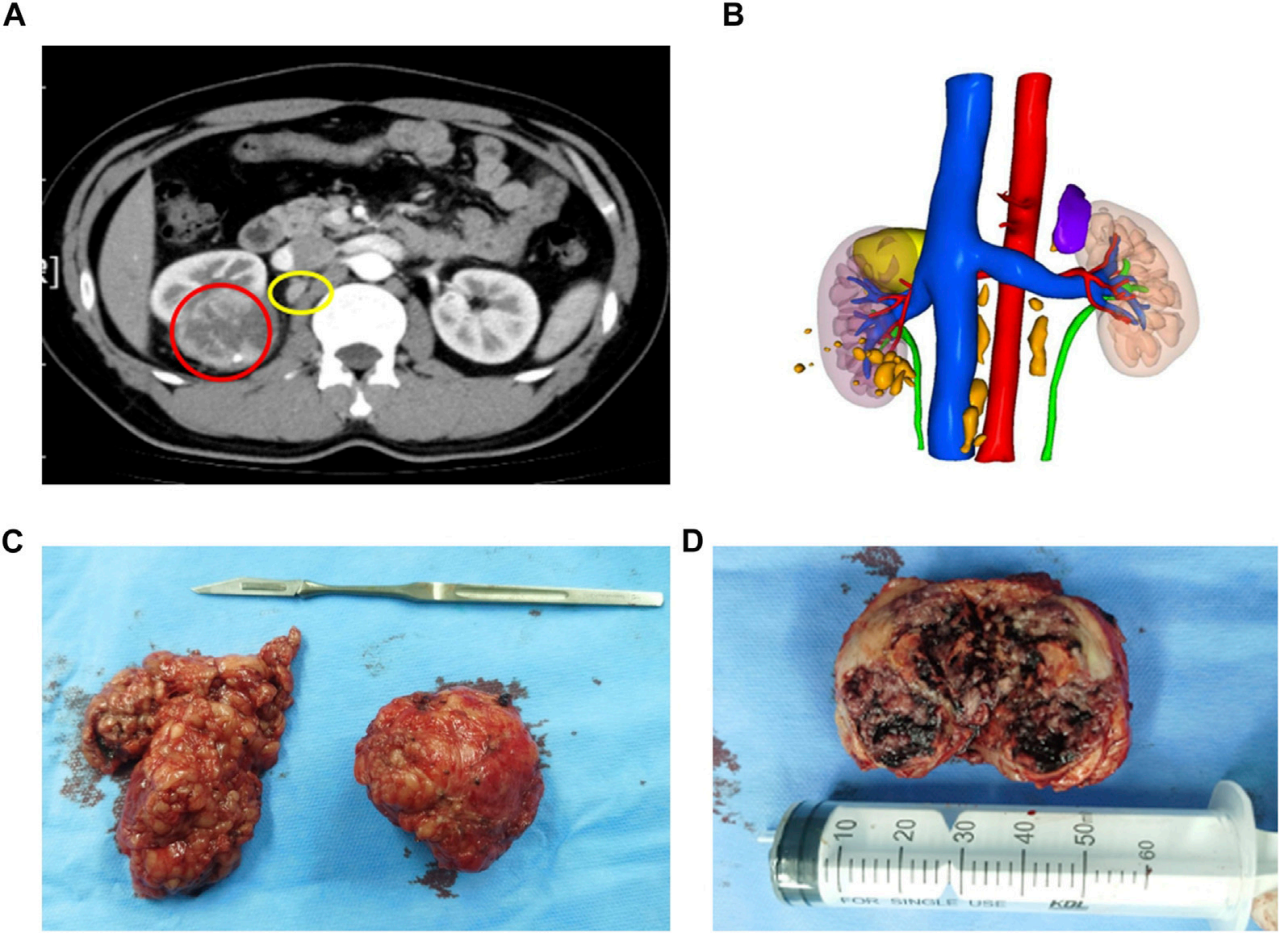
Conventional CT and 3D reconstruction demonstrated a tumor and enlarged lymph nodes. **(A)** Abdominal CT scan detected a solid mass (6 cm in diameter) at the right kidney (red circle) and multiple enlarged lymph nodes (yellow circle). **(B)** 3D reconstruction of the urinary system which includes lesions (tumor: yellow; enlarged lymph nodes: orange), kidneys, collective system, and blood vessels. **(C)** A well-circumscribed solid mass on the right and multiple enlarged hilar lymph nodes scavenged on the left. **(D)** Cut surface of a well-encapsulated tumor lesion showing gray-white or gray-yellow and a large area of necrosis, and the tumor was 6 × 5.5 × 5 cm^3^ in size.

The patient was a 32-year-old man who manifested fever and right flank paroxysmal blunt pain. No other special clinical symptom was identified. Computed tomography (CT) showed an inhomogeneous mass, 6 cm in diameter, in the right kidney, and multiple lymph nodes enlargements were found adjacent to inferior vena cava and abdominal aorta (Figures 1A,B) and no evidence of distant organ metastasis. The patient had no past medical history of malignancy. Glomerular filtration rate (GFR) of the right kidney was 49 ml/min/1.73 m^2^, and 68 ml/min/1.73 m^2^ on the left, which showed decreased kidney function on the right side (Webster et al., 2017). As there were no contraindications to surgery, the patient then underwent right PN and scavenging of enlarged lymph nodes by laparoscopic surgery on 9 Oct 2020. Intraoperatively, a well-circumscribed solid mass, 6 cm in diameter, was identified in the upper part of the right kidney and multiple hilar and inferior vena cava lymph nodes were scavenged (Figure 1C). The cut surface of the tumor was gray-white or gray-yellow with a large area of necrosis (Figure 1D).

**FIGURE 2:**
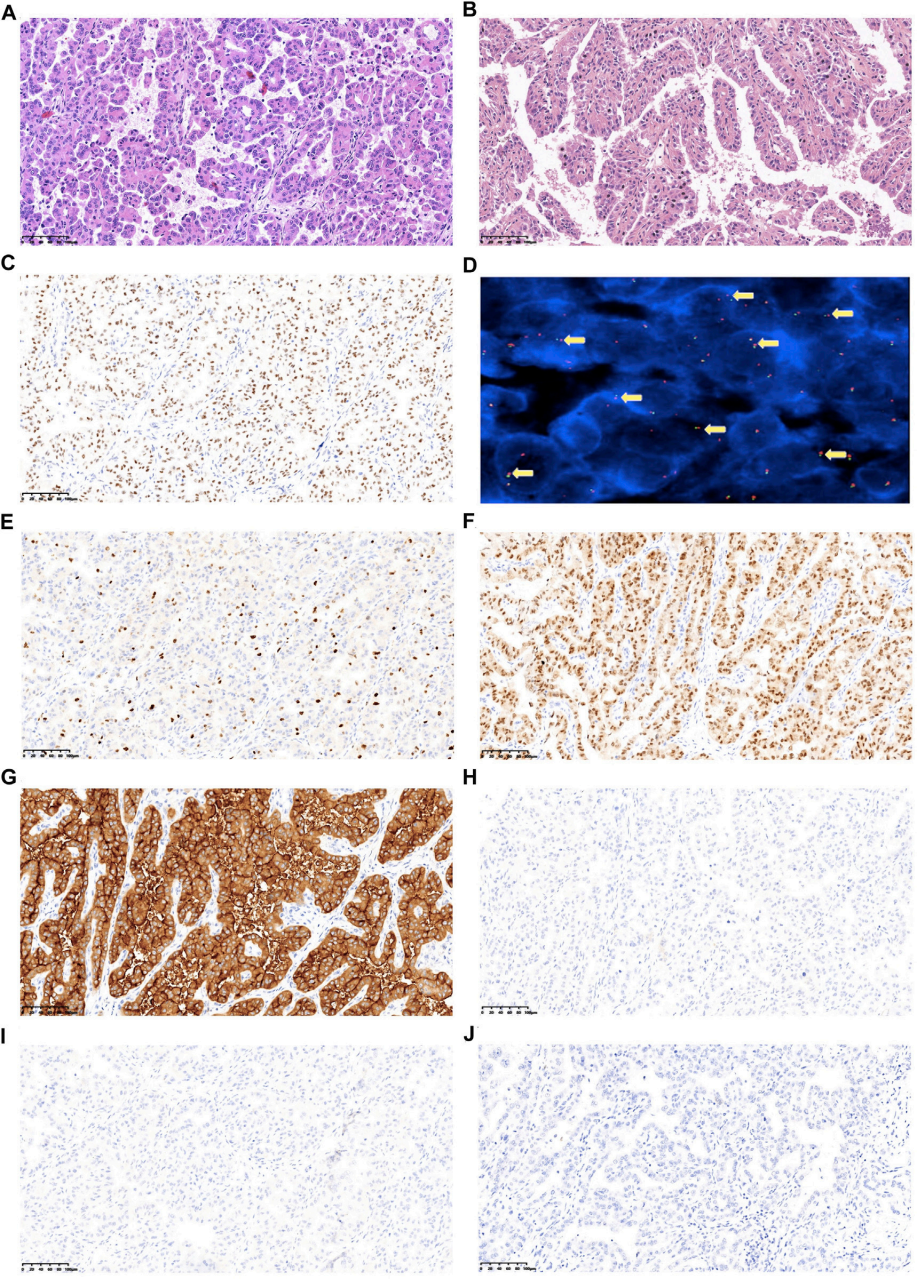
Representative images of the postoperative pathological features of the analyzed tumors. **(A)** HE (hematoxylin and eosin) staining of tumor sample, 200x. **(B)** HE staining of metastatic hilar lymph nodes sample, 200x. **(C)** Tumor cells display TFE3 nuclear positive. **(D)** TFE3 break-apart probe assay identified split signals, 1,000x. **(E)** Ki67 expression. **(F)** PAX-8 expression. **(G)** CD10 expression. **(H)** CD117 expression. **(I)** CA9 expression; **(J)** HMB45 expression (Images E-J have a magnification at 200x).

Postoperative pathological examination revealed that the case was considered to be Xp11.2 translocation/TFE3 gene fusions associated with RCC, with a size of 6 × 5.5 × 5 cm^3^, and the right renal hilar lymph node was confirmed with metastases (Figures 2A,B). The tumor observed was in pT1bN1M0, stage III according to AJCC Cancer Staging Manual, presenting WHO/ISUP Grade 3 of nuclear grade. Immunohistochemistry (IHC) results revealed positive for TFE3, almost the entire neoplastic cell nuclei stained positive for TFE3, with moderate (2+) to strong (3+) staining intensity (Figure 2C). FISH results showed a separated red and green signal in most nuclei of the tumor cells, which indicated the rearrangement of the TFE3 gene (Figure 2D). Immunohistochemical staining showed positive for Ki67 (15%), PAX-8, and CD10 (Figures 2E–G), and negative for CD117, CA9, and HMB45 (Figures 2H–J).

**FIGURE 3:**
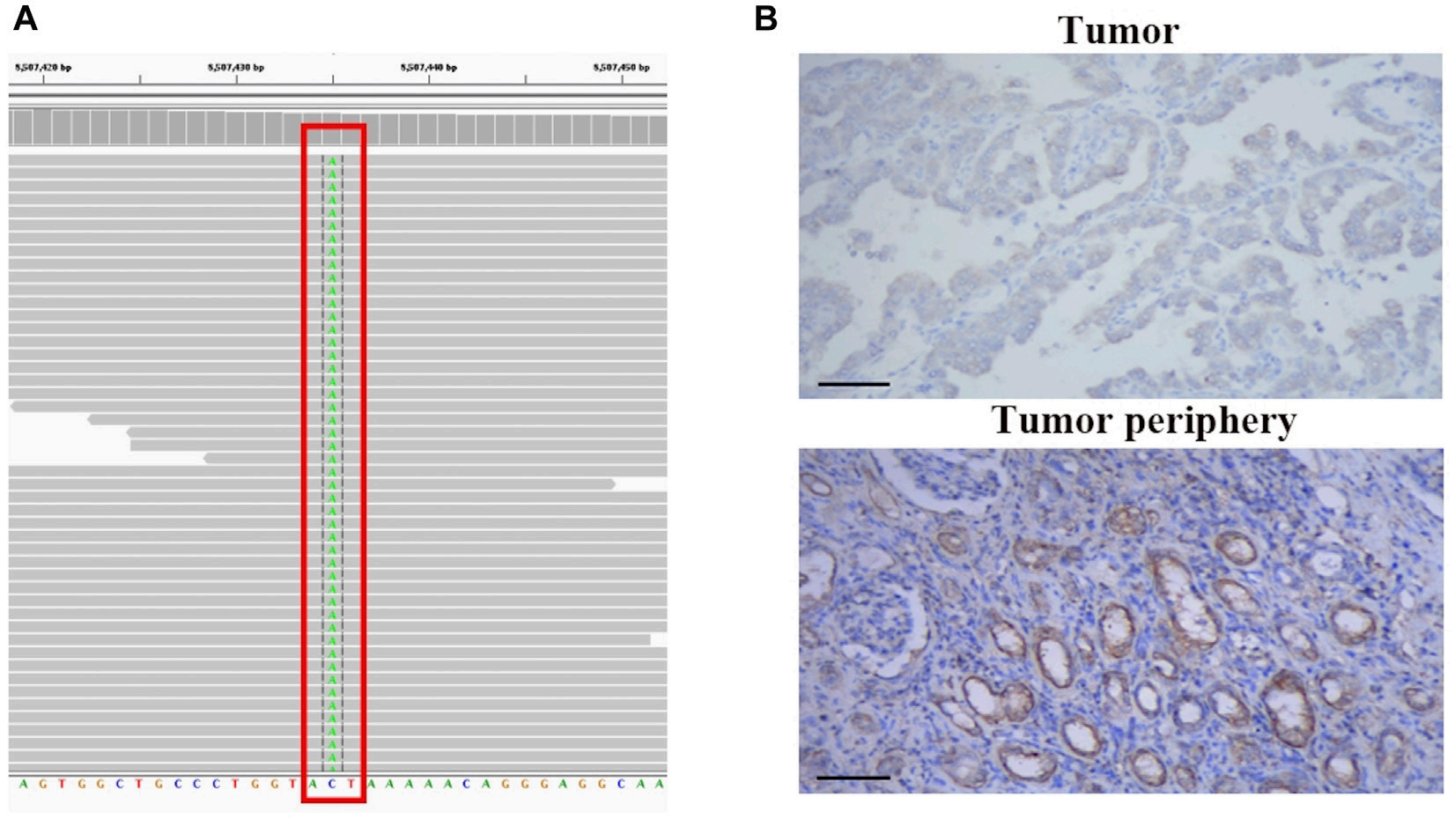
NGS-based identification of *PTPRD* mutation and PTPRD expression of the analyzed tumors. **(A)** NGS-based identification of the c.1544-1G>T mutation. **(B)** PTPRD expression in tumor sample (upper part) and tumor periphery (lower part), 200x.

Next, we performed genomic profiling with a multi-gene next-generation sequencing (NGS) panel (Onco Panscan™, Genetron Health) to identify any genetic alterations that might be relevant to the prognosis of this tumor. A splice site mutation c.1544-1G>T of protein tyrosine phosphatase receptor delta (*PTPRD*) was detected, which is localized in the splice acceptor site of intron 21 of the *PTPRD* gene. Sequencing results are presented in Figure 3A. We suppose this *PTPRD* c.1544-1G>T variant could probably impair *PTPRD* splicing and cause premature termination of protein transcription. And then, we performed IHC of anti-PTPRD by using a tumor sample. Weak PTPRD expression was confirmed in the tumor sample (Figure 3B, upper part) compared to the tumor periphery (Figure 3B, lower part). Previous studies have reported that the inactivation of PTPRD can promote angiogenesis and metastasis and is associated with a high malignant phenotype (Solomon et al., 2008; Bae et al., 2019). Then, we could predict the aggressive behavior of this tumor. Therefore, we decided to perform postoperative adjuvant therapy to treat the remaining enlarged lymph node adjacent to the abdominal aorta and to prevent the progression of the tumor.

**FIGURE 4:**
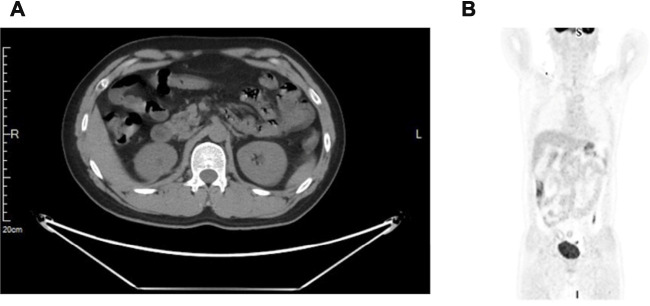
A positron emission tomography-computed tomography (PET/CT) scan. **(A)** No suspicious primary lesion recurrence in the right kidney after surgery. **(B)** Neither lymph nodes enlargement adjacent to the aorta nor remote organ metastases.

Regarding the results from several clinical studies, combination immunotherapy with target therapy is superior to monotherapy in the treatment of advanced renal cancer. KEYNOTE-426, a phase 3 clinical trial, showed superior efficacy of immune checkpoint inhibitor (pembrolizumab) plus tyrosine kinase inhibitor (axitinib) over sunitinib monotherapy in treatment-naive, advanced renal cell carcinoma (Rini et al., 2019; Powles et al., 2020; Qu et al., 2021). Then, after 2 weeks of surgery, the patient was given a novel anti-PD-1 antibody (camrelizumab, 200 mg) intravenously once every 3 weeks, 16 cycles for about 1 year plus axitinib (5 mg) orally twice daily for 1 year. Mild adverse events (grade 1–2) were only observed in the 1st–2nd cycle of treatment, which includes periodontitis, abdominal distension, and mild to moderate diarrhea. During the rest cycles of the treatment, no other treatment-related adverse events were observed. The patient underwent periodic follow-up examinations, including laboratory tests and CT every 3 months. PET-CT was performed on the 12th month postoperatively, which indicated neither lymph node enlargement adjacent to the aorta nor remote organ metastases (Figures 4A,B), indicating a clinical complete response. Until now, the patient is currently alive without recurrence or metastasis 18 months after surgery and the GFR of the right kidney was 39 ml/min/1.73 m^2^, 58 ml/min/1.73 m^2^ on the left, which showed most preservation of kidney function. This patient is still on further follow-up without medication.

## Discussion

Xp11.2 tRCC typically affects children, with a one-third incidence in juveniles and 1%–1.6% incidence in adults (Bruder et al., 2004; Sukov et al., 2012; Kmetec and Jeruc, 2014). Xp11.2 tRCC in adult patients may be associated with advanced stages, large tumors, and may present with metastatic disease with poor prognosis (Qiu et al., 2010; Qu et al., 2016). As Xp11.2 tRCC has no specific macroscopic appearance, and clinically most tumors resemble clear cell RCC (Armah and Parwani, 2010). Unlike pediatric RCC, cytogenetics is not routinely performed for adult RCC due to the relatively lower incidence rate, which results in most misdiagnoses as conventional RCC. The diagnosis of Xp11.2 tRCC relies on morphological features, IHC findings, and molecular pathological analysis (Calio et al., 2019). TFE3-IHC has the advantages of rapid diagnosis, economy, and high sensitivity in diagnosing Xp11.2 tRCC. Thus, TFE3-IHC can be performed proactively as a screening test, and FISH can be performed as a verification test. Hirobe et al. suggested that the combination of TFE3-IHC and FISH is an effective method to diagnose Xp11.2 tRCC, which can improve specificity and potentially eliminate false positives resulting from overstaining (Hirobe et al., 2016). In this case, the results of positive immunostaining of TFE3 and positive TFE3 break-apart FISH assay, as well as positive CD10 and negative CD7, CD117, led to the ultimate diagnosis of Xp11.2-TFE3 tRCC (Calio et al., 2019).

*PTPRD* mutation was detected by NGS and reduced PTPRD expression was confirmed in tumor cells in this study. PTPRD belong to the protein tyrosine phosphatases family and function as a tumor suppressor in human cancers (Zhao et al., 2015). Cox et al. provided the first evidence that PTPRD was identified as a frequent target of deletion in human cancers (Cox et al., 2005). Subsequent studies further showed somatic mutations of *PTPRD* were identified in colorectal cancer, lung cancer, glioblastoma, and melanoma (Sjoblom et al., 2006; Weir et al., 2007; Solomon et al., 2008; Veeriah et al., 2009). Inactivation of *PTPRD* was linked to malignancy in glioma and melanoma (Solomon et al., 2008; Ortiz et al., 2014). Deleterious mutations of *PTPRD* are associated with bad prognosis in lung cancer patients (Sun et al., 2021a). Furthermore, with regard to protein expression, reduced PTPRD expression was found in tumor tissue in several cancers including renal cancer which was associated with a bad prognosis (Li et al., 2011; Bae et al., 2019). On the contrary, overexpression of PTPRD suppressed colon cancer cell migration, suggesting that gain of PTPRD could suppress tumor metastasis (Veeriah et al., 2009). The evidence seen is consistent with the notion that PTPRD normally functions as a tumor suppressor. Our case found a splice site mutation c.1544-1G>T of *PTPRD*, which probably impairs *PTPRD* splicing and causes premature termination of protein transcription. And then reduced PTPRD expression was displayed in the tumor sample compared to the tumor periphery (Figure 3B). Based on the aforementioned evidence, we assumed this case may associate with a poor prognosis.

Current treatment for Xp11.2 tRCC is still controversial. The treatment recommended for Xp11.2 tRCC is mostly based on small retrospective studies and similar to conventional RCC. Surgical intervention plays an important role and is currently focusing on organ preservation; however, this is based upon tumor localization and institutional experience (He et al., 2015; Calio et al., 2019). Adult patients at an advanced stage had poorer outcomes even with postoperative adjuvant monotherapy (He et al., 2015; Liu et al., 2016). Choueiri et al. reported that only three patients (20%) could achieve a partial response when treated with anti-angiogenesis targeted therapy, which appeared to demonstrate some efficacy for adult patients with metastatic Xp11.2 RCC (Choueiri et al., 2010). Immune checkpoint inhibitors (ICI) represent a novel class of drugs used in metastatic RCC, however, experience in advanced Xp11.2 tRCC is limited. Clark et al. reported that patients with tRCC patients showed better outcomes when treated with ICI than those treated with anti-angiogenesis therapy (Clark et al., 2019). In a retrospective analysis by Boilève et al., the median PFS for tRCC patients during the first ICI treatment was only 2.5 months (range, 1–40 months), four patients experienced a partial response (16.7%), and 3 (12.5%) had stable disease (Boileve et al., 2018). However, in a case represented by Masago et al., immunotherapy by a combination of nivolumab plus ipilimumab was ineffective for metastatic Xp11.2 tRCC patients, and during the drug administration period, pleural effusion occurred (Masago et al., 2020). Furthermore, studies showed that *PTPRD* inactivation could promote angiogenesis, and *PTPRD* mutation could benefit from immunotherapy in lung cancer (Bae et al., 2019; Sun et al., 2021b; Wang et al., 2021). Therefore, we assume antiangiogenic therapy in combination with ICI may be superior to monotherapy for this patient.

Camrelizumab, a humanized monoclonal antibody that selectively blocks the binding of PD-1 to PD-L1 and eventually inhibits the immune escape of tumor cells, has shown activity across a wide range of malignant carcinomas (Mo et al., 2018). Qu et al. reported the results of camrelizumab plus famitinib in an advanced or metastatic RCC cohort regardless of lines of prior systemic therapy. This phase II basket study showed total objective response rate (ORR) was 60.5%, and in treatment-naive patients, the ORR reached 84.6% (95% CI, 54.6%–98.1), which showed potent and enduring antitumor activity in patients with advanced or metastatic RCC (Qu et al., 2021). Most common grade 3 or four treatment-related adverse events included proteinuria (18.4%), hypertension (18.4%), neutropenia (13.2%), palmar-plantar erythrodysesthesia syndrome (10.5%), and hypertriglyceridemia (10.5%). No treatment-related deaths occurred, and no new safety signals were observed in this study (Qu et al., 2021). Also, in KEYNOTE-426, a phase 3 clinical study showed superior efficacy of ICI plus axitinib over sunitinib monotherapy in treatment-naive advanced RCC (Rini et al., 2019; Powles et al., 2020). Costs and risks considered we choose camrelizumab plus axitinib as a treatment for this patient. Until now, adverse events were only observed in the first two cycles of treatment. The patient is currently alive without recurrence or metastasis 18 months after surgery, indicating a clinical complete response.

The limitation of this study is that longer follow-up will be required to assess the long-term effect.

In summary, the occurrence of Xp11.2 tRCC is rare. Immunohistochemical and cytogenetic findings allow the differential diagnosis of Xp11.2 tRCC. *PTPRD* mutation might be a favorable indicator for Xp11.2 tRCC patients managed by PN and followed by the adjuvant therapy of combined immune checkpoint inhibitor and tyrosine kinase inhibitor. Due to the poor prognosis in adult Xp11.2 tRCC, more research is needed to evaluate the effects of postoperative adjuvant therapy.

## Data Availability

The original contributions presented in the study are included in the article/supplementary materials, further inquiries can be directed to the corresponding authors.
